# Impact of Persistent Medication Adherence and Compliance with Lifestyle Recommendations on Major Cardiovascular Events and One-Year Mortality in Patients with Type 2 Diabetes and Advanced Stages of Atherosclerosis: Results From a Prospective Cohort Study

**DOI:** 10.5334/gh.1273

**Published:** 2023-11-01

**Authors:** Evgeniya V. Shalaeva, Arjola Bano, Ulugbek Kasimov, Bakhtiyor Janabaev, Markus Laimer, Hugo Saner

**Affiliations:** 1 Graduate School for Health Sciences, University of Bern, Bern, Switzerland; 2 Tashkent Medical Academy, Tashkent, Uzbekistan; 3 School of Medicine, Central Asian University, Tashkent, Uzbekistan; 4 Institute for Social and Preventive Medicine, University of Bern, Bern, Switzerland; 5 Department of Cardiology, Inselspital, Bern University Hospital, University of Bern, Bern, Switzerland; 6 Clinic for Diabetology, Endocrinology, Nutrition and Metabolism, University Hospital Bern, Bern Switzerland

**Keywords:** medication adherence, lifestyle compliance, type 2 diabetes, peripheral artery disease, atherosclerosis, cardiovascular disease prevention, mortality

## Abstract

**Background:**

The aim of this study was to evaluate the impact of single and combined effects of persistent medication adherence and compliance with lifestyle recommendations on the incidence of major adverse cardiovascular events (MACE) and one-year all-cause mortality in patients with type 2 diabetes (T2D) and peripheral artery disease (PAD) after partial foot amputation (PFA), representing a unique cohort of patients with advanced stages of atherosclerosis.

**Methods:**

This is a prospective cohort study of 785 consecutive patients (mean age 60.9 ± 9.1 years; 64.1% males). Medication adherence was evaluated by using the proportion of days covered (PDC) measure calculation and was defined as a PDC ≥80%. It derived as an average of PDCs of the following four classes of drugs: a) antidiabetics (oral hypoglycemic medications and/or insulin); b) ACEI or ARBs; c) Statins; d) antiplatelet drugs. Lifestyle compliance was defined as a PDC ≥80% comprising of PDCs of a) physical activity of ≥30 minutes per day; b) healthy nutrition and weight management; c) non-smoking. Cox proportional hazard models adjusted for confounders were used.

**Results:**

Total all-cause mortality was 16.9% (n = 133) at one-year follow-up. After adjusting for confounders, compared to adherent/compliant patients (n = 432), non-adherent and/or non-compliant patients had an increased risk of one-year mortality: HR = 8.67 (95% CI [5.29, 14.86] in non-adherent/non-compliant patients (n = 184), p < 0.001; HR = 3.81 (95% CI [2.03, 7.12], p < 0.001) in adherent/non-compliant patients (n = 101) and HR = 3.14 (95% CI [1.52, 6.45] p = 0.002) in non-adherent/compliant patients (n = 184). The incidence of MACE followed similar pattern (HR = 9.66 (95% CI [6.55, 14.25] for non-adherence/non-compliance; HR = 3.48 (95% CI [2.09, 5.77] and HR = 3.35 (95% CI [1.89, 5.91], p < 0.001 for single adherence or compliance.

**Conclusions:**

Medication adherence and compliance to lifestyle recommendations have shown to be equally effective to reduce the incidence of MACE and one-year mortality in patients with diabetes and PAD after PFA representing a population with highly advanced stages of atherosclerotic disease. Our findings underline the necessity to give lifestyle intervention programs a high priority and that costs for secondary prevention medications should be covered for patients under these circumstances.

**Lay Summary:**

- This study analyzed the single and combined effects of medication adherence and compliance with lifestyle recommendations on cardiovascular events and mortality in patients with type 2 diabetes and advances stages of atherosclerosis over a period of one year.
- Evaluation of medication adherence included antidiabetics, statins, dual antiplatelets and ACEI/ARBs, whereas lifestyle recommendations included healthy nutrition, physical activity and smoking cessation.
- Persistent medication adherence and lifestyle changes have shown to be equally effective to reduce the incidence of MACE and one-year mortality in patients representing a population with highly advanced stages of atherosclerotic disease, and positive effects added up to a double effect if patients were persistently adherent and compliant with both interventions.

## Introduction

Patients with type 2 diabetes (T2D) and advanced stages of atherosclerosis are at a very high risk of major cardiovascular events (MACE) and mortality [1,2]. Type 2 diabetes is a chronic metabolic disease which leads not only to significant medical costs but also early mortality or disability because of an increased risk for the development of macrovascular and microvascular complications [3,4,5]. The global prevalence of diabetes is approximately 12% and is expected to rise by 5% every five-years, mainly due to the increased lifespan and prevalence of obesity [6]. In accordance, diabetes complications are expected to increase disproportionally affecting vulnerable populations with lower social and economic factors [7]. These circumstances may be encountered more often in countries with lower socioeconomic status, where patients do not have equitable access to preventive medicine, early diagnosis, treatment and advanced healthcare services. These patients are more likely to seek medical help at more advanced disease stages with persisting complications.

In Uzbekistan, according to the latest Word Health Organization (WHO) data published in 2020 prevalence of T2D is estimated to be 9.1% of the population of 33 million. Death from T2D in Uzbekistan reached 6,205 or 3.84% of total deaths and was the fourth leading cause of death after cardiovascular disease (42.19%), stroke (13.4%) and liver disease (5.93%). Death from MACE (coronary artery disease (CAD) and stroke) accounted for a total of 56.53% of all-cause mortality ranking Uzbekistan third in the world [8,9].

The lifetime prevalence of diabetic foot ulcer (DFU) is over 33%, and is increasing [3]. Diabetic foot ulcer commonly develops in middle-aged patients due to a long duration of T2D and poor control of blood glucose levels [10]. A foot ulcer is the initial event in more than 85% of major amputations that are performed in patients with diabetes [11]. Peripheral arterial disease (PAD), present in half of patients with DFU, is an independent predictor of limb loss and excess mortality [11]. A recent review shows that during the last decades there has been a decline in the incidence of total lower limb amputations, which may be explained by the declines in major amputations such as transfemoral amputation (TFA). Smaller relative declines have been reported for minor amputations, whereas some low and middle income countries have reported increases [12,13]. Overall trends suggest that there may be a relative increase in the number of minor amputations like partial foot amputations (PFA) being performed in the clinical setting to prevent TFA [12]. This is supported by a recent systematic review showing that five-year mortality after the incidence of PFA was ranging from 52% to 82% and for TFA from 53% to 100% and did not change through decades regardless of advanced treatment availability [1].

Type 2 Diabetes patients with advanced atherosclerotic disease are typically on numerous medications for secondary and tertiary prevention [14]. Medication adherence and healthy lifestyle are the most important factors to improve patients’ health [15]. The Centers for Disease Control and Prevention (CDC) estimates that non-adherence to medication prescription causes 30–50% of chronic disease treatment failures, 20–30% of new prescriptions are never filled at the pharmacy and medication is not taken as prescribed 50% of the time [16]. Non-adherence estimates a 25% increase in all-cause mortality [16]. Previous observational and randomized controlled studies typically focused on one specific adherence intervention. Thus, the one-year adherence to oral antihyperglycemic medication for adults with T2D, measured as ≥80% proportion of days covered (PDC) was associated with a 10% reduction in one-year all-cause mortality HR, 0.90 [95% CI, 0.82–0.99] [17]. High adherence to statins was significantly associated with decreased risk of all-cause death, MACE or stroke, compared to low adherence (HR = 0.61; 95% CI, 0.54–0.71; p < 0.001) [18]. One-year statin exposure in T2D high-risk patients was associated with a 25% risk reduction in all-cause mortality [19]. Non-compliance to antihypertensive medications was associated with increased all-cause mortality (HR = 1.45; 95% CI, 1.36–1.54) as compared to the highest adherence. Optimizing lifestyle has been shown to reduce the risk of many adverse health outcomes and risk factors in all ages and both sexes. Main components of a healthy lifestyle are physical activity, healthy nutrition and non-smoking [15]. A recent analysis showed that behavior and lifestyle factors, such as smoking, unhealthy nutrition and lack of physical activity may be attributed to 16% to 65% of all-cause premature mortality [20,21].

However, it is currently still not clear to what extent persistent compliance to lifestyle recommendations and adherence to the best available medication including ACEI/ARBs, statins, antiplatelet treatment and antidiabetics may contribute to improve intermediate and long-term outcomes in patients with highly advanced stages of atherosclerotic disease.

The aim of this study was to evaluate the impact of single and combined effects of persistent medication adherence and compliance with lifestyle recommendations on incidence of MACE and one-year all-cause mortality in patients with type 2 diabetes and peripheral artery disease after partial foot amputation representing a unique cohort of patients with advanced stages of atherosclerosis.

## Methods

Overall, 853 T2D patients, with advanced stages of atherosclerosis, documented PAD without previous history of major or minor limb amputations and unhealed DFU complicated by gangrene and/or infection of the toe(s)/foot, underwent partial foot amputation (PFA) at the Republican Centre of Purulent Surgery and Complications of Diabetes, Tashkent Medical Academy, Tashkent, Uzbekistan. All patients were enrolled in the prospective observational cohort study. After hospitalization, patients were referred to the state outpatient clinics (branches of the Republican Center) based on patients’ physical address where patients’ medical charts, events and prescriptions have been monitored. Of the overall 853 patients with the incidence of PFA, 68/853 patients could not be contacted beyond one month after surgery (mostly international patients), and have been excluded from the analysis, 785 patients with one-year follow-up data are included in the final analysis.

Baseline characteristics were retrieved at the hospital admission including patient’s risk factors such as obesity, hypertension, history of coronary artery disease (CAD), PAD, history of diabetes as well as socio-economic factors and lifestyle risk factors such as self-reported smoking status, physical activity and nutrition. Diabetes was defined by a glycated hemoglobin HbA1C ≥ 6.5%, history of physician based diagnosis or use of anti-diabetic medications according to 2019 ESC Guidelines on diabetes, pre-diabetes and cardiovascular diseases [22]. Smoking has been defined according to the NHIS/CDC concept [23]. Coronary artery disease was defined according to the 2019 ESC Guidelines for the diagnosis and management of chronic coronary syndromes [24]. Obesity was defined by a BMI ≥ 30 kg/m [25]. Blood pressure (BP) was measured after some rest, in sitting position, at the beginning and at the end of the healthcare providers exam in both arms, the mean of two measurements was used [26,27,28]. Hypertension was defined according to guidelines of the Eighth Joint National Committee (JNC8) [26,29].

Forefoot ulcer and/or gangrene were classified as stage C (ischemia) or stage D (ischemia and infection), and grade 2–3 (deep and very deep) according to the Texas Wound Classification or grade 4 and 5 according to Wagner – Meggitt’s classification [30].

### Treatment protocol

Within 24 hours of admission, we followed a specific protocol for the preparation of patients for surgery, including normalization of arterial and central venous pressure, adequate diuresis, electrolyte and acid-base balance and body temperature. We provided adequate intravenous fluids, antibacterial and antifungal drugs, antidiabetic medications (including insulin when indicated) and antithrombotic and anticoagulant therapy. We immediately started prescribed medication including atorvastatin 80 mg daily (starting preoperatively), clopidogrel, first day 300 mg, then 75 mg daily, antihypertensive therapy (including drugs from either ACE or ARB groups), and treatment of co-morbidities if indicated; aspirin 75–150 mg daily started three days after amputation for continuous use (minimum one year). There was no death during and within seven days after operation in the surgical hospital.

During hospitalization, patients and relative caregivers (after patients’ permission) had physician consultations and educational sessions on secondary prevention recommendations, including healthy nutrition (therapeutic diet for Pevzner, Table 9, diabetes [31]) and weight management, physical activity adapted to the situation after PFA, smoking cessation programs, diabetes management, blood pressure control, thrombosis preventions and lipid-lowering medication.

During in-hospital stay, patients were assessed for medication adherence and compliance to lifestyle recommendations daily. Patients also received a starter package containing educational leaflets, details of a free phone patient helpline and website, labels with a reminder to take medication of study interest, a diary (paper-based or electronic), and a calendar with detailed instructions of time, dosage, and duration of prescribed treatment, and diaries for registering drug intake, physical exercise, and diet.

After discharge, nurses conducted phone calls 2–3 times per week during first months and every two weeks during the one-year follow-up. Physicians had phone conversations if indicated to assess how patients followed recommendations to complete diaries. Physician visits were scheduled every week during the first month after discharge and every month during the following 12 months period at the diabetes center. Every three months patients’ data was also collected from outpatient clinics at the place of residence.

Patients and close relatives also received regular personalized text messages and phone calls throughout the study. At every examination, patients and caregivers were asked to present their diaries with daily drug intake, redeemed prescriptions, and blisters of prescribed medications. Visit data was compared with the nurses’ diaries communication. If the patient did not take the drugs as prescribed, we searched for reasons, conducted interviews and provided training to improve adherence.

The costs for diabetes treatment, including in-hospital surgery for amputations and all emergency services are included in the Guaranteed Benefits Package and covered by the government. Diabetes medications and ACEI/ARBs refills were made free of charge for patients at the outpatient clinics based on the patient’s residence and, at the same time, were also assessed to control for adherence. Nevertheless, patients had to cover costs for all other medications including antihypertensives, statins, antiplatelets and were asked to keep pharmacy receipts and medication boxes.

### Evaluation of medication adherence

Medication adherence has been evaluated by using the proportion of days covered PDC measure calculation. Adherence to medication was defined as a PDC ≥80% [32,33]. PDC has been calculated for each patient by using the following steps: *Step 1*: The patient’s treatment period was defined as a number of days from the prescription date (typically the day of admission to the hospital) to the end of the enrollment (one-year follow-up), disenrollment or death. In the current study, we did not have a loss of follow-up unless the patient death. If the prescription was discontinued by the physician or changed to another generic, this was carefully noted and measures were adjusted; *Step 2*: Within the treatment period, we counted the days a patient was covered by each of the following four classes of drugs: a) antidiabetics (oral hypoglycemic medications and/or insulin); b) ACEI or ARBs; c) Statins; d) antiplatelet drugs. In case a single drug product was changed to a combination product containing at least two single drugs or when there is an overlap of a combination product with another combination product, the days covered per each medication were assessed individually. The doses of medications were adjusted during patient visits depending on blood glucose, blood pressure, LDL-cholesterol, coagulogramme data and limb complications, respectively. The changes in prescription (types of medications and doses) were adjusted per each individual patient. *Step 3:* The number of covered days found in *Step 2* was divided by the number of days found in *Step 1* per each medication, then this number was multiplied by 100 to obtain the PDC (as a percentage) for each group of drugs. *Step 4*: As all 785 patients had prescriptions of four groups of targeted medications, we summed five PDCs and divided them by four to receive an overall mean adherence rate per each individual patient. *Step 5*: If the patient reached a mean 80% PDC, the patient was considered as adherent to medications [32,33].

### Evaluation of compliance with lifestyle recommendations

The proportion of days covered PDC measure calculation has also been used to evaluate compliance with lifestyle recommendations. Compliance was defined as a PDC ≥80%. Proportion of days covered was calculated for each patient by using the following steps: *Step 1*: The patient’s treatment period was defined as a number of days from the first day of admission to the hospital to the end of the enrollment (one-year follow-up), or death. *Step 2:* Within the treatment period we counted the days the patient was following lifestyle recommendations: a) physical activity of ≥30 minutes per day (as patients typically could not use the operated foot approximately six months after surgery while the wound was healing, walking with crunches, breathing, and balance exercises to build body strengths to support themselves as well as special exercises for the affected extremity were recommended for at least 30 minutes per day); b) healthy nutrition (therapeutic diet table no. 9, Pevzner, for diabetes) [31] with at least five servings of vegetables/fruits per day with weight management [34]; c) non-smoking. In cases of occasional/rare smokers, the number of non-smoking days during follow-up was calculated. If no contraindications, patients’ physical rehabilitation was started on day 1 after PFA. *Step 3*: The number of covered days found in *Step 2* was divided by the number of days found in *Step 1* per each lifestyle changes recommendation, then we multiplied this number by 100 to obtain the PDC (as a percentage). *Step 4*: If a patient continued smoking regardless of compliance with nutrition and physical activity, he/she was considered non-compliant to lifestyle recommendations. In case of quitting smoking, the PDC was calculated as a mean PDC measure of nutrition and physical activity: (PDC nutrition + PDC physical activity)/2. In the case of occasional/rare smokers, the PCD was calculated as non-smoking days/patients’ treatment period*100. The following formula was used to calculate the overall PDC for lifestyle recommendations: (PCD smoking + PDC nutrition + PDC physical activity)/3. *Step 5*: If the patient reached an overall mean 80% PDC in *step 4*, the patient was considered compliant with lifestyle change recommendations.

Depending on compliance/adherence results, patients were divided into four groups: Group 1 includes non-adherent to medications and non-compliant with lifestyle changes patients (for both components, overall PDC in study cohort was <40%). Group 2 includes patients who did not show lifestyle changes compliance PDC was <80%, ranging of 43–62% of days measured but were adherent to medications regimen ≥80% during one follow-up year, Group 3 includes patients who did not meet medication adherence criteria PDC <80%, ranging 47–73%, but were compliant to lifestyle recommendations ≥80% PDC, and Group 4 includes patients who were both persistently medications adherent and lifestyle changes compliant (≥80% PDC for both components).

### Cardiovascular outcomes and all-cause mortality

The primary clinical end-point in this study was all-cause mortality and MACE. One-year all-cause was defined as a composite endpoint of death from any cause one-year after partial foot amputation that was performed in the hospital. Follow-up postoperative outcomes were collected by scheduled appointments, telephone call, patients’ charts and confirmation by social death certificate.

Major adverse cardiovascular events were included as secondary outcome and was defined as a composite endpoint as cardiovascular death, nonfatal myocardial infarction (MI), stent thrombosis, acute stroke, or unstable arrhythmia [35]. Acute stroke was diagnosed based on clinical presentation and non-contrast brain CT [35,36]. Cardiovascular death was defined as death due to MI, congestive heart failure, stroke, or arrhythmias or any unknown causes of death not explained by non-cardiac etiologies. Diagnosis of MI was confirmed by two of three findings: chest pain or equivalent symptom complex; positive cardiac biomarkers; ECG changes typical of MI [35,37]. In patients who had multiple cardiac events, only the first one was counted toward MACE to create the MACE-free Kaplan-Meyer curve.

### Literature search strategy

We used Search strategy MEDLINE (Ovid) reflecting the research question indicated in the title. Research question: ‘Does medication adherence and compliance with lifestyle recommendations improve outcomes (major cardiovascular events and one-year mortality) in patients with type 2 diabetes and peripheral artery disease and/or advanced stages of atherosclerosis.’

We reviewed single or combined effects of the 4 classes of drugs: a) antidiabetics (oral hypoglycemic medications and/or insulin); b) ACEI or ARBs; c) Statins; d) antiplatelet drugs. Lifestyle compliance was focused on a) physical activity of ≥30 minutes per day; b) healthy nutrition and weight management; c) non-smoking. Articles closely related to our population of interest were selected to include in the current manuscript.

### Statistical analysis

Statistical analyses were performed using SPSS software (v27, IBM, Chicago, IL, USA). Descriptive statistics for studied variables are presented as mean ± SD (standard deviation) for normally distributed continuous variables, median with interquartile range for non-normally distributed continuous variables and frequency with percentage for categorical variables. Variables were compared with independent Student t tests for normally distributed continuous data, and Chi-square test for categorical data. Differences between groups were determined by a one-way analysis of variance (ANOVA), with a subsequent Tukey’s/Dunnet C post hoc test.

Time to event was calculated as the period between PFA and death or MACE. Unadjusted Kaplan–Meier curves for MACE-free and all-cause mortality free survival were created depending on medication adherence and lifestyle compliance groups using the log-rank test (Mantel-Cox) along with Breslow (Generalized Wilcoxon) and Tarone-Ware tests for the period from the partial foot amputation during the one-year follow-up.

The association of adherence and compliance with the risk of all-cause mortality and incident MACE was assessed using Cox proportional hazard models. Analyses were adjusted for age, gender, HbAC1, history of diabetes and diabetes-associated complications (diabetic nephropathy, angiopathy, neuropathy), severity of PAD (ankle-brachial pressure index) level of PFA, presence of foot infection, cardiovascular comorbidities (e.g. CAD, history of myocardial infarction and heart revascularization, congestive heart failure), arterial hypertension, obesity, behavioral factors (smoking status, nutrition, etc.), revised cardiac risk index at admission. A p-value of <0.05 was considered statistically significant. Variables used in the analysis did not have missing data. Cox Proportional Hazard assumption was checked and met.

A sensitivity test of the COX regression analysis was conducted to present the adjusted effect of compliance to lifestyle recommendations and medications adherence on incidence of MACE and all-cause mortality. Lifestyle compliance PDC ≥80% and medication adherence PDC ≥80% were considered as exposure and coded as a binary variable ‘1’ vs. PDC <80% as ‘0’. Analyses were adjusted for potential confounders.

## Results

Mean age of the total cohort of 785 patients was 60.9 ± 9.1 years, 64.1% were males. PAD diagnosis was documented in all participants with an ankle-brachial pressure index ranging between 0.63 and 0.89. Great toe amputation was performed in 227 patients (28.9%), one toe or a combination of 2–5 toes in 366 patients (46.6%), and trans-metatarsal amputation in 192 patients (24.4%). At admission, 87.6% of patients had HbA1c levels ≥8; 20.6% of patients were on statins; 29.2% on anti-hypertensive treatment and 51.9% had a history of aspirin use. Concerning smoking status, 43.6% of patients were never or ex-smokers, 13.4% occasional smokers, 28.8% smoked <1 pack/day and 14.3% ≥1 pack/day. 10.4% of study participants had normal weight, 54.2% were overweight and 35.4% were obese. Arterial hypertension was diagnosed in 63.6% of patients, dyslipidemia in 72.9%. Coronary artery disease was diagnosed in 276 patients (63.9%), of which 178 had a history of myocardial infarction (Table 1).

**Table 1:** Baseline characteristics of 785 patients with type 2 diabetes undergoing surgery for partial foot amputation.


BASELINE VARIABLE	TOTAL COHORT,	NON-COMPLIANT/NON-ADHERENT	NON-COMPLIANT/ADHERENT	COMPLIANT/NON-ADHERENT	COMPLIANT/ADHERENT	p-VALUE

	n = 785	n = 184	n = 101	n = 68	n = 432	

Age, years mean ± SD	60.9 ± 9.1	62.0 ± 10.1	61.3 ± 9.0	61.7 ± 9.1	60.3 ± 8,6	0.138

Sex, n (%)						<0.001

Male	503 (64.1)	128 (69.5)	86 (85.1)	20 (29.4)	269 (62.3)	

Female	282 (35.9)	56 (30.4)	15 (14.8)	48 (70.6)	163 (37.7)	

HbAC1, % mean ± SD	11.4 ± 2.7	12.3 ± 2.8	11.5 ± 2.7	11.0 ± 2.5	11.2 ± 2.5	<0.001

Diabetes duration, years, mean ± SD	7.9 ± 5.9	8.5 ± 6.4	7.4 ± 5.7	9.5 ± 6.2	7.6 ± 5.8	0.098

New diagnosed diabetes, n (%)	54 (6.8)	14 (7.6)	7 (6.9)	5 (7.4)	28 (6.4)	0.774

Diabetic nephropathy, n (%)	175 (22.3)	56 (30.4)	19 (18.8)	14 (20.5)	86 (19.9)	0.030

Oral anti-diabetes drugs, n (%)						

Biguanide monotherapy: metformin	145 (18.4)	36 (19.6)	15 (14.8)	17 (25.0)	77 (17.8)	

Sulfonylureas monotherapy: gliclazide, glimepiride	127 (16.2)	23 (12.5)	16 (15.8)	12 (17.7)	76 (17.6)	

Combination of metfomin with sulfonylureas or DDP4 (sitagliptin)	235 (29.9)	49 (26.6)	32 (31.7)	14 (20.6)	140 (32.4)	

SGLT-2i or GLP-1-mimetics	–	–	–	–	–	

Insulin, n (%)	224 (28.5)	62 (33.7)	31 (30.7)	20 (29.4)	111 (25.7)	0.207

Insulin with metformin	186 (23.7)	49 (26.6)	25 (24.8)	15 (22.1)	97 (22.5)	

BMI, mean ± SD	28.7 ± 4.1	28.6 ± 3.9	29.8 ± 8.0	28.4 ± 3.1	28.8 ± 3,5	0.131

Normal weight, n (%)	101 (12.9)	26 (14.1)	14 (13.9)	16 (23.5)	45 (10.4)	

Overweight n (%)	427 (54.4)	104 (56.5)	56 (55.4)	33 (48.5)	234 (54.2)	

Obesity, n (%)	257 (32.7)	54 (29.3)	31 (30.7)	19 (27.9)	153 (35.4)	

Arterial hypertension, n (%)	498 (63.4)	112 (60.9)	73 (72.3)	38 (55.9)	275 (63.6)	0.161

Stage 2, n (%)	448 (57.1)	105 (57.1)	63 (62.4)	32 (47.1)	248 (57.4)	

Stage 1, n (%)	50 (6.4)	7 (3.8)	10 (9.9)	6 (8.8)	27 (6.2)	

Elevated, n (%)	162 (20.6)	30 (16.3)	12 (11.9)	17 (25.0)	103 (23.8)	

Normal, n (%)	125 (15.9)	42 (22.8)	16 (15.4)	13 (19.1)	54 (12.5)	

Dyslipidemia, n (%)	560 (71.3)	132 (71.7)	65 (64.3)	48 (70.6)	315 (72.9)	

History statin use, n (%)	162 (20.6)	36 (19.5)	23 (22.8)	15 (22.1)	88 (20.4)	0.732

History aspirin use, n (%)	408 (51.9)	69 (37.5)	54 (53.5)	31 (45.6)	254 (58.8)	

Smoking, n (%)	443 (56.4)	145 (78.8)	96 (95.0)	13 (19.1)	189 (43.7)	<0.001

Never/past smoker, n (%)	342 (43.6)	39 (21.2)	5 (4.9)	55 (80.9)	243 (56.2)	0.137

Occasional	105 (13.4)	29 (15.8)	6 (5.9)	5 (7.3)	65 (15.0)	

<1 pack/day	226 (28.8)	71 (38.6)	56 (55.4)	5 (7.3)	94 (21.7)	

≥1 pack/day	112 (14.3)	45 (24.5)	34 (33.7)	3 (4.4)	30 (6.9)	

Coronary artery disease, n (%)	516 (65.7)	134 (72.8)	60 (59.4)	46 (67.6)	276 (63.9)	0.083

Congestive heart failure	260 (33.1)	87 (47.2)	25 (24.7)	24 (35.3)	124 (28.7)	0.003

Previous myocardial infarction, n (%)	178 (22.7)	75 (40.8)	24 (23.8)	19 (27.9)	60 (13.9)	0.145

Revised cardiac risk index, n (%)						0.029

II	169 (21.5)	26 (14.1)	28 (27.7)	12 (17.6)	103 (23.8)	

III	224 (28.5)	49 (26.6)	25 (24.7)	24 (35.3)	126 (29.2)	

IV	392 (49.9)	109 (59.3)	48 (47.5)	32 (47.1)	203 (47.0)	


Every patient was followed over one year after PFA was performed. There was no loss of follow-up unless patient death (mean follow-up time 0.912 ± 0.24 years). At one-year, 184 patients (23.4%) did not persistently follow medication regimen as prescribed and did not follow lifestyle changes recommendations described above and were identified as non-adherent and non-compliant (Group 1). The PDC for every patient in this group fell below 40%. They either continued smoking, used regularly unhealthy nutrition (confections, sugar beverages, food rich of fat and carbohydrates and required increased dosages of insulin or antidiabetic medications), did not properly use or did not use at all dual antiplatelets and statin therapy, did not maintain blood pressure on targeted levels <130/80 mm Hg, or did not follow recommendations for regular physical activity. Notably, 753 (95.9%) patients regularly followed anti-diabetic tablet treatment as prescribed. One-hundred one patients were identified as medication adherent (PCD ≥80%) but were non-compliant to lifestyle recommendations (Group 2), 68 patients were compliant to lifestyle recommendations (PCD ≥80%) but non-adherent (Group 3). Four-hundred thirty-two patients (55.0%) were identified as both compliant and adherent meeting criteria as described in the methods section (Group 4). They followed a diet as prescribed, stopped smoking (if smoker), increased physical exercises to ≥30 min/day, and had >80% drug intake as prescribed.

Baseline characteristics of patients of the entire cohort and within each adherent/compliant group are shown in Table 1. Groups were not different in regard to age, race, social-economic status or level of education. Non-compliance and non-adherence were associated with single or divorced marital status and living alone. Patients from compliant/non-adherent group were mostly women (70.6%), had lower prevalence of obesity and hypertension, had higher proportion of no/never smokers, compared to the other groups (Table 1). Systolic and diastolic blood pressure levels, HbAC1 values, prevalence of coronary artery disease, congestive heart failure and diabetes nephropathy was greater in the non-adherence and non-compliance group. Patient groups were not different in regard to the level of amputation (toes vs metatarsal).

### All-cause mortality

There was no in-hospital mortality up to seven days after minor foot surgery. Thirty-days all-cause mortality in the entire cohort was 32/785 (4.1%), and one-year all-cause mortality was 16.9% (n = 133). All-cause mortality was 7.6% (41/536) in patients with good medication adherence compared to 36.9% (92/249) in non-adherent patients (Chi-square = 103.7; p < 0.001). Among lifestyle compliant patients, All-cause mortality was 7.0% (35/499) in patients with good compliance with lifestyle recommendations compared with 34% (98/286) in non-compliant patients (Chi-square = 95.9, p < 0.001).

Within 30 days after discharge from the hospital after minor foot amputation, 32/785 patients (4.1%) died, 27 of them were non-compliant with lifestyle recommendations and non-adherent to medication regimen representing 84.3% of all deaths of the cohort (Pearson Chi-Square = 69.28, p-value < 0.001) (Table 2). one-year mortality among non-compliant/non-adherent patients was 42.9% (79/184) and was the highest among all groups. All-cause mortality among non-compliant/adherent patients was 19.8% (20/101), among compliant/non-adherent was 17.6% (12/68), and the lowest 5.1% was among both persistently compliant and adherent patients (22/432 patients); Pearson Chi-Square = 132.056, p-value < 0.001 (Table 2).

**Table 2:** Clinical outcomes across categories of medication adherence and lifestyle recommendations compliance in 785 patients with type 2 diabetes after partial foot amputation.


VARIABLE, n (%)	NON-COMPLIANT/NON-ADHERENT n = 184	NON-COMPLIANT/ADHERENt n = 101	COMPLIANT/NON-ADHERENT n = 68	COMPLIANT/ADHERENT n = 432	TOTAL COHORT, n = 785

Postoperative all-cause mortality, n (%)					

30 days	27 (14.7)	0	1 (1.5)	4 (0.9)	32 (4.1)

1-year	79 (42.9)	20 (19.8)	12 (17.6)	22 (5.1)	133 (16.9)

Postoperative major adverse cardiovascular events, n (%)					

30 days	51 (27.7)	8 (7.9)	3 (4.4)	8 (1.8)	70 (8.9)

1-year	116 (63.0)	28 (27.7)	19 (27.9)	37 (8.6)	200 (25.5)


Multivariable Cox regression analysis was conducted to examine the association of persistent compliance and adherence with the risk of all-cause mortality. After adjusting for confounders, compared to adherent/compliant patients, all other groups had an increased risk of one-year mortality. In non-adherent/non-compliant patients, we found HR = 8.67 (95% CI [5.29, 14.86], p < 0.001; in adherent/non-compliant patients HR = 3.81 (95% CI [2.03, 7.12], p < 0.001), in non-adherent/compliant patients HR = 3.14 (95% CI [1.52, 6.45] p = 0.002) (Table 3).

**Table 3:** Multivariable adjusted Cox regression analysis for the association of persistent compliance and adherence with the risk of all-cause mortality and major adverse cardiovascular events in type 2 diabetes patients undergoing partial foot amputations.


ALL-CAUSE MORTALITY	HR	95.0% CI		

		LOWER	UPPER	p-VALUE

Group adherence and compliance (reference)				0.000

Compliance/adherence (ref)	1			

Non-compliance/adherence	3.807	2.033	7.127	<0.001

Compliance/non-adherence	3.138	1.521	6.145	0.002

Non-compliance/non-adherence	8.670	5.299	14.187	<0.001

**Major adverse cardiovascular events**				

Compliance/adherence (ref)	1			<0.001

Non-compliance/adherence	3.479	2.097	5.772	<0.001

Compliance/non-adherence	3.350	1.899	5.910	<0.001

Non-compliance/Non-adherence	9.663	6.554	14.245	<0.001


Analyses were adjusted for age, gender, history of diabetes and diabetes-associated complications, severity of PAD, level of PFA, cardiovascular comorbidities, arterial hypertension, obesity, revised cardiac risk index, Charlson comorbidity score.

**Figure 1:**
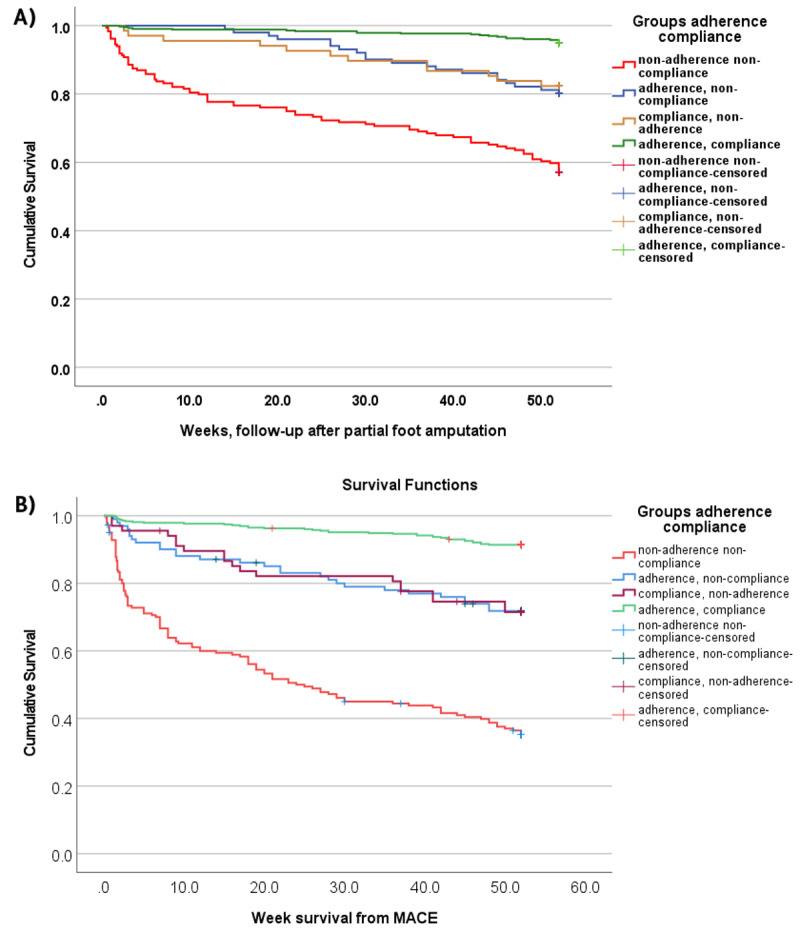
**Unadjusted all-cause mortality free and major adverse cardiovascular events (MACE) – free Kaplan-Meier survival curves in patients with type 2 diabetes undergoing partial foot amputation A) all-cause mortality B) MACE**. Patients were divided into 4 groups: persistently adherent/compliant (n = 432), adherent/non-compliant (n = 101), compliant/non-adherent (n = 68), non-adherent/non-compliant (n = 184).

One-year all-cause mortality-free Kaplan-Meier survival curve grouped by medication adherence and lifestyle compliance are shown in Figure 1A.

A sensitivity test of the COX regression analysis was conducted to present the adjusted effect of compliance to lifestyle recommendations and medications adherence to all-cause mortality. Following lifestyle recommendations and being adherent to medications was associated with a decrease in all-cause mortality (HR = 0.46, 95% CI 0.27–0.73, p = 0.001 for lifestyle and HR = 0.35, 95% CI 0.22–0.55 for medication adherence, p < 0.001, respectively).

MACE was the main contributor to death in these patients. Thus, during 30 days after minor foot amputation, 70/785 patients (8.9%) developed MACE, of them 51 patients were non-compliant and non-adherent to medication regimen representing 72.9% of all MACE in the cohort (Table 2). one-year incidence of MACE of the entire cohort was 200/785 (25.5%). Non-compliant/non-adherent patients experienced MACE in 63.0% cases (116/184), with the highest rate among all groups. Major adverse cardiovascular events among non-compliant/adherent patients was 27.7% (28/101), and was similar to compliant/non-adherent patients, 27.9% (19/68), and the lowest 8.6% was among both persistently compliant and adherent patients (37/432 patients); Pearson Chi-Square = 202.328, p-value < 0.001 (Table 2). During one-year follow-up MACE occurred in 63/535 patients who were medication adherent compared to 137/249 non-adherent patients (Chi-square = 167,2, p < 0.001), whereas MACE occurred in 55/498 patients who were compliant with lifestyle recommendations compared to 145/286 non-compliant patients (Chi-square = 150.3, p < 0.001).

One-year MACE Kaplan-Meier survival curve grouped by the medication adherence and lifestyle changes compliance are shown in Figure 1B. After adjusting for confounders, compared to adherent/compliant patients, all other groups had an increased risk of incidence of MACE (Table 3). In non-adherent/non-compliant patients we found HR = 9.663 [95% CI 6.55, 14.245], p < 0.001; in adherent/non-compliant patients HR = 3.479 (95% CI [2.09, 5.77], p < 0.001), and in non-adherent/compliant patients HR = 3.35 (95% CI [1.89, 5.91], p < 0.001).

## Discussion

### Single and combined effect of adherence and compliance on MACE and mortality

To our best knowledge, this is the first large prospective cohort study of patients with type 2 diabetes, PAD and advanced stages of atherosclerosis, with a main focus on persistent patients’ adherence to medication and compliance with lifestyle recommendations. Our results indicate that if patients were both adherent to medication and compliant with lifestyle recommendations (>80% proportion of time), there was an 88% reduction in the risk of all-cause mortality compared to non/adherent and non/compliant patients. Persistent adherence to the best available treatment including antidiabetics, lipid lowering medications, antithrombotic treatment and antihypertensives was associated with a 56.5% risk reduction even when patients` compliance with lifestyle recommendations was <80% of time. Being only compliant with lifestyle recommendations over 80% of time was associated with a significant benefit in decreasing the risk of all-cause mortality by up to 65.5%.

Overall, both interventions for medication adherence and lifestyle changes compliance had similar effects even in patients with advanced stages of atherosclerotic disease, and positive effects add up to a double effect if patients were persistently adherent and compliant. Problems with adherence to medication in our study were mostly related to the complexity of the medication regime, dosage and frequency, forgetfulness, inability to buy medications for continuous use, adverse effects, or combinations of these factors.

Because we could not find previous publications with a throughout evaluation of the components of adherence to medication and lifestyle modifications, we can only summarize the results from previous publications as interventions. Thus, one-year adherence to oral antihyperglycemic medication for adults with T2D, measured as PDC ≥80% showed a 10% reduction in one-year all-cause mortality [17]. One-year statin exposure in T2D high-risk patients was associated with a 25% risk reduction in all-cause mortality [19]. Another study showed an approximately 39% risk decrease of all-cause mortality, MACE, or stroke in patients newly treated with statins who had high adherence (PDC ≥80) compared with low adherence to statins [18]. Non-compliance to antihypertensive medications was associated with an 1.45 increased risk of all-cause mortality as compared to the highest adherence [15]. Amstrong and co-authors showed that patients with PAD who received aspirin, statin medications and ACEIs and who did not smoke at the baseline of diagnostic or interventional lower-extremity angiography had a 36% decreased risk of MACE and a 44% reduction in the risk of three-years mortality compared to patients receiving less than four of the mentioned therapies [38]. Other studies showed that smoking, unhealthy nutrition and lack of physical activity are attributed to 16% to 65% of all-cause premature mortality [20,21].

The majority of patients die from major adverse cardiovascular events (MACE) as a consequence of CAD and advanced atherosclerosis in other vascular sites [39]. Our study also showed that patients with T2D PAD who underwent PFA have a very high risk for MACE and all-cause mortality which is not inferior compared with patients undergoing TFA. In our previous work, we observed a tenfold risk increase of MACE for non-compliant patients after trans-femoral (major) amputations, who are considered to have higher risk of cardiovascular complications compared to patients with minor foot amputations [40]. Therefore, interventions to prevent cardiovascular complications are usually considered to be of a lower priority in patients who underwent PFA. Our new finding is that patients undergoing minor foot amputation are also at very high risk for MACE and mortality which is not inferior compared to the risk in patients after major limb amputations and therefore indicates a strong need for multidisciplinary approaches and interventions to improve short-term prognosis and survival in these patients. This includes measures to improve medication adherence and compliance with lifestyle recommendations.

Of note, at baseline only 162/785 (20.6%) of our patients met international guidelines recommendations at study start [41], including non-smoking status, use of antidiabetics, statins; anti-hypertensive and antiplatelet drugs. Similar to our study, Iacopi and co-authors showed that among 1,315 patients with DFU, only 24% were prescribed all five guideline recommended therapies [42].

### Study strengths and limitations

A major strength of our study is the size of the cohort with T2D, PAD and highly advanced stages of atherosclerosis from one single institution with no loss of follow-up. This is to our best knowledge the first prospective cohort study with a main focus on the combined effect of maintaining persistent patients’ adherence to medication and compliance with lifestyle recommendation using mathematical calculations of Proportional Days Covered. The simultaneous impact of persistent adherence to four classes of medications (antidiabetics, ACEI or ARBs; statins and antiplatelet drugs), non-smoking status, healthy nutrition and weight management and physical activity (of ≥30 minutes per day) on one-year mortality and the incidence of MACE was assessed adjusting for multiple potential confounders.

However, interpretation of our results is limited to patients with similar characteristics. The specific setting of patients, institutions and treatment modalities in Uzbekistan must be considered when comparing our results with findings from other populations.

Our study cannot draw conclusions about possible benefits of compliance and adherence over longer time periods. Although adherence to medication has been extensively evaluated, we may have missed some medication failures. Compliance with lifestyle recommendations has been evaluated based on self-reporting which has well known limitations and therefore my overestimate true lifestyle changes. Finally, the possibility of residual confounding cannot be excluded given the observational study design.

## Conclusions

Medication adherence and compliance to lifestyle recommendations have shown to be equally effective to reduce the incidence of MACE and one-year mortality in patients with diabetes and PAD after PFA representing a population with highly advanced stages of atherosclerotic disease. Our findings underline the necessity to give lifestyle intervention programs a high priority and that cost for secondary prevention medications should be covered for patients under these circumstances.
